# Salivary Microbiome Variation in Early Childhood Caries of Children 3–6 Years of Age and Its Association With Iron Deficiency Anemia and Extrinsic Black Stain

**DOI:** 10.3389/fcimb.2021.628327

**Published:** 2021-03-23

**Authors:** Rui Han, Jin Yue, Haozhi Lin, Nan Du, Jinfeng Wang, Shuting Wang, Fanzhi Kong, Jiaying Wang, Wei Gao, Lei Ma, Shoushan Bu

**Affiliations:** ^1^ Department of Stomatology, The First Affiliated Hospital of Nanjing Medical University, Nanjing, China; ^2^ Department of Stomatology, The Affiliated Hospital of Qingdao University, Qingdao, China; ^3^ Chinese National Human Genome Center, Beijing, China; ^4^ Beijing Institutes of Life Science, Chinese Academy of Sciences, Beijing, China

**Keywords:** early childhood caries (ECC), salivary microbiome variation, iron deficiency anemia (IDA), extrinsic black stain (BS), children 3-6 years of age

## Abstract

ECC is a common clinical manifestation of the oral cavity in childhood and Iron deficiency-anemia (IDA) is a high-risk factor but extrinsic black stain on the tooth surface is a protective factor for caries. There is limited information about oral microecological change in early children who suffer from ECC with IDA and extrinsic black stain (BS). This study enrolled 136 children aged 3-6 years. Dental caries and teeth BS were examined. Saliva was collected for 16S rRNA gene and fingertip blood were for Hemoglobin test. There are 93 ECC including 13 with IDA (IDA ECC) and 80 without IDA (NIDA ECC) and 43 caries free (CF) including 17 with BS (BSCF) and 26 without BS (NBS CF). Statistical analysis of microbiota data showed differences of the oral flora in different groups. The oral flora of the IDA ECC group had a high diversity, while the BSCF group had a low diversity. The bacterial genera Bacillus, Moraxella, and Rhodococcus were enriched in the IDA ECC while Neisseria was enriched in the NIDA ECC. Neisseria only exhibited high abundance in the BSCF, and the remaining genera exhibited high abundance in the NBSCF. Interestingly, the BSCF had the same trend as the NIDA ECC, and the opposite trend was observed with IDA ECC. We established random forest classifier using these biomarkers to predict disease outcomes. The random forest classifier achieved the best accuracy in predicting the outcome of caries, anemia and black stain using seven, one and eight biomarkers, respectively; and the accuracies of the classifiers were 93.35%, 94.62% and 95.23%, respectively. Our selected biomarkers can achieve good prediction, suggesting their potential clinical implications.

## Introduction

Early childhood caries (ECC) is the most common chronic disease in childhood (2016b), and it is defined as decay involving the primary dentition in children less than 72 months of age and is a main reason for which young children go to hospitals (Sheller et al., 1997), and prosthodontic procedures are often performed under general anesthesia because of the extent of tooth decay and the young ages of the children affected (2016b; Schroth and Smith, 2007). The distribution of dental caries is uneven in the population; 81% of caries were found to occur in 26% of children (Chen et al., 2019).

Alarmingly, children with iron deficiency anemia have a higher rate caries than the general population of children (Deane et al., 2018). Tang R S and other scholars found that ECC in children is often accompanied by malnutrition and think that anemia is strongly related to ECC (Tang et al., 2013). In the author’s previous epidemiological survey, the hemoglobin was significantly lower in children with caries than children without caries, similar to findings of Schroth et al. (2013) and Bansal et al. (2016).

Extrinsic black stain(BS) clinically appears in some children as black line stains on the tooth neck (1/3) or pigmented dark lines parallel to the gingival margin. This black tooth stain is an extrinsic discoloration, and it always occurs in both primary and permanent dentition. This stain is difficult to remove and easy to reappear after removal (Hattab et al., 1999), It is reported the incidence of black tooth stains ranges from 1% to 20%, showing the same distribution in both genders (Żyła et al., 2015). However there are different reports about the effect of extrinsic black stain on ECC (SUN et al., 2013; Chen et al., 2014; Tripodi et al., 2016; WU et al., 2018). Black staining of teeth in children has been associated with lower dental caries (Garan et al., 2012; de Rezende et al., 2019).

The oral cavity is the entrance to the gastrointestinal tract, and its micro-ecological status has been reported to be associated with the incidence of dental diseases such as dental caries (Ling et al., 2010). The caries are not only caused by several specific bacteria in which *Streptococcus mutans* is the most important cariogenic bacteria, but related to the changes in the overall oral microbiome. The microflora of extrinsic black stain on teeth is dominated by *Actinomyces* spp. and has lower cariogenic potential than non-discolored dental plaque. The oral microbiome of children with ECC and caries-free (CF) children and the imbalance of microecology in the pathogenesis of dental caries were reported (Lamont et al., 2018), whether the children IDA damage oral micro ecological balance cause caries deserve further research. Especially the importance of the role of the microbiome in extrinsic BS and caries formation in primary dentition, while information regarding the role of the oral microbiota in IDA ECC and extrinsic BS-related CF oral cavity colonization is still limited. This study set up a cohort of children aged 3-6 years, divide into ECC and CF two groups, further into IDA ECC and NIDA ECC two subgroups, BSCF and NBS CF two subgroups in order to research the salivary microbiome variation in early childhood caries of children and its association with iron deficiency anemia and extrinsic black stain.

## Materials and Methods

### Study Population

A total of 1584 preschool children aged 3-6 years from 14 kindergartens in Shandong Qingdao city, China, were investigated from 2017 to 2018. A total of 136 children were enrolled in our study according to the following inclusion criteria: 1) the child’s parent signed the informed consent form; 2) no abnormal physical examination result was observed for half a year before sample collection; 3) the child was willing to cooperate with the oral examination; 4) saliva samples were successfully collected and stored; 5) 0.1 mL of blood from the fingertips was successfully collected; 6) the 16S gene was successfully extracted and amplified; and 7)there was no antibiotic intake in the 3-month period before sample collection. The ethical review of this study was approved by the Institutional Review Board of the Affiliated Hospital of Qingdao University.

### Examination of Dental Caries and Extrinsic Black Stain in Teeth

We used mouth mirrors to check the oral conditions of the children. If we suspected that caries were present, a community periodontal index (CPI) probe was used to assist in the inspection. No radiography was performed. The caries examination method and criteria were as recommended by the World Health Organization (WHO, 1997).A trained and standardized dentist performed all the dental examinations. Duplicate examinations were performed on 7 randomly selected subjects to assess intra-examiner reliability. Dental caries were detected at the cavitation level. The diagnostic criteria for caries-free teeth were with no fillings due to caries, and a complete crown without signs of caries. The pre-caries stage and similar early caries were excluded: such as caries on the adjacent surface of deciduous teeth, white spot lesion changes or enamel demineralization, discolored or rough spots on the crown use a disposable probe to detect the softening of the sensory tissue. None of the above can be included in the record of caries free (CF).

The extrinsic BS on teeth is firmly attached to the surface of the tooth as black dots. It is difficult to remove by brushing. In addition, a linear discoloration line is generally formed parallel to the gingival margin, covering at least one-third or more of two different teeth of the clinical dental crown (Koch et al., 2001; Heinrich-Weltzien et al., 2009). According to the standards specified by Gasparetto et al. (2003), BS is divided into mild pigmentation (marked 1), moderate pigmentation (marked 2) and severe pigmentation (marked 3). There are melanin dots or discontinuous black thin lines parallel to the gingival margin for mild pigmentation (marked 1); there are clear and continuous black thin lines, and the range does not exceed 1/3 of the neck in moderate pigmentation (marked 2); there are clear and continuous black thin lines, and the range is more than 1/3 of the tooth neck in severe pigmentation (marked3); and no such characteristic is observed when there is no pigmentation (marked 0). In this context, patients with mild pigmentation (marked 1), moderate pigmentation (2) and severe pigmentation (3) were included in the extrinsic BS samples.

To ensure the accuracy of our inspection data, we checked the reliability of our examiner, and all measurements were reviewed for 10% of the participants. Cohen’s kappa (κ) test was used to assess the intra-examiner reliability, and our examiner’s kappa (κ) values were all greater than 0.9.

### Routine Fingertip Blood Test and Iron Deficiency Anemia Diagnosis

*Whole blood* (0.1 mL) was collected from the fingertips of each child in an EDTA anticoagulation tube. The routine blood test was performed by a Mindray-B5300 automated blood analyzer. According to the anemia diagnostic criteria from the World Health Organization, when the hemoglobin content of children aged 5 to 59 months is less than 110 g/L or that of children aged 5 to 11 years old is less than 115 g/L, the condition is diagnosed as anemia (WHO, 2018). An IDA diagnosis was given if 2 of the following 3 blood parameters were abnormal: 1. hemoglobin (< 110 g/L); 2. serum ferritin (< 15 ng/L); and 3. MCV (< 80 fL), MCH<27pg, MCHC <310 g/L (Scholl and Reilly, 2000).

### Saliva Collection and DNA Extraction

Before saliva collection, the children were asked not to eat any food, drink or brush their teeth for 2 h. Each child was instructed to spit saliva into a sterile container until a 2 ml unstipulated complete saliva sample was collected. Sampling was performed by two dental examiners. During saliva collection, aseptic precautions were strictly required. Attention was given to placing the saliva collection tube inside the lips to collect saliva and to the removal and placement of the test tube cover. Upon contamination, a new test tube was used to prevent interference by other flora and ensure reliability of the experimental results. The collected samples were quickly frozen on dry ice, transported to the laboratory within 2 h, and then stored at -80°C until use (Quinque et al., 2006). DNA was extracted using the Genomic DNA Kit (TIANamp version number:DP180123).

### Polymerase Chain Reaction Amplification of 16S rRNA Genes and MiSeq Sequencing

Polymerase chain reaction (PCR) amplification of the V3–V4 region was performed with the following primers containing Illumina adapter sequences and dual-index barcodes to tag each sample: 341F 5′-CCTACGGGNGGCWGCAG-3′ and 805R 5′-GACT ACHV GGGT ATCT AATCC-3′. The PCR conditions were as follows: 95°C for 3 min, followed by 25 cycles of denaturation at 95°C for 30 s, annealing at 65°C for 30 s, extension at 72°C for 30 s and a final extension step at 72°C for 5 min. PCR products were then cleaned using AMPure XP Beads (item no. A63882, Beckman Coulter, Inc., CA, USA). The amplicon sequencing libraries were constructed in accordance with the 16S Metagenomic Sequencing Library Preparation protocol (Illumina, Inc., San Diego, CA, USA). Paired-end sequencing with a read length of 250 bp was performed on a MiSeq Instrument (Illumina, Inc., San Diego, CA, USA) using a MiSeq v2 Reagent Kit (Illumina, Inc., San Diego, CA, USA).

### Processing and Statistical Analysis of Microbiota Data

Sequencing data were processed using the QIIME package(version 1.9.1) (Caporaso et al., 2010) as follows. Raw reads were demultiplexed and quality filtered, allowing no N characters and a maximum unacceptable Phred quality of Q20. Chimeric sequences were detected and removed using USEARCH algorithms (Edgar and Flyvbjerg, 2015). The remaining sequencing reads were assigned to operational taxonomic units (OTUs) with a 97% similarity threshold and subsequently picked by UCLUST against a closed reference table, the latest version(v13.8) of the Greengenes OTU database (Edgar, 2010). Alpha diversity of OTU libraries was described using the Chao1, Shannon, Simpson, phylogenetic diversity (PD) whole tree, and observed species. Distance matrices were constructed using the unweighted UniFrac algorithms in QIIME from the whole community phylogenetic tree. Microbiota dissimilarity was depicted by principal coordinate analysis (PCoA). We used PICRUSt1.0.0 (Phylogenetic Investigation of Communities by Reconstruction of Unobserved States) (Langille et al., 2013) to predict metagenome function from the 16S rRNA data and a reference genome database(GreenGenes-v13.5) with default parameters. The Wilcoxon-Mann-Whitney test with adjustments according to Benjamini-Hochberg(FDR) was conducted to detect differences in two groups using R 3.4.0. A P value < 0.05 was considered statistically significant after adjusting for FDR.

### Linear Discriminant Analysis Effect Size Analysis and Random Forest Classifier

We used the LDA effect size (LEfSe) tool to identify the oral microbial communities in different groups, indicating the types of potential microbial biomarkers for different conditions (Segata et al., 2011). First, the previously generated OTU table was filtered, and then, the potential noise caused by low-abundance taxa was eliminated by including taxa present in 1% of the sample with at least 5% abundance. Next, using the Kruskal-Wallis test, α<0.1, and LDA score larger than 2.0, LefSe analysis was performed to determine the discriminant characteristics.

The randomForest (version 4.6-14) package of R language was used to build the random forest classifier. We filtered biomarkers with LDA scores larger than 2 and used sequential forward selection to select the best feature set. We first evaluated the accuracy for each feature and obtained the feature with the largest accuracy as the starting point for the feature selection process. Then, for the other features, we added one feature each time to evaluate the accuracy of the classifier. Then, we added the feature with the largest accuracy to the classifier. We repeated this process until the largest accuracy of the classifier to the remaining features was less than that of the previous round. To evaluate the performance of each feature or feature set to the classifier, we used fivefold (ECC vs CF) or leave-one-out (IDA ECC vs NIDA ECC and BS CF vs NBS CF) cross-validation and repeated this process 100 times.

Considering the imbalance of the number of samples in the two groups, we also treated the samples by oversampling and undersampling. The details are as follows: For oversampling, we resampled the smaller group with replacement and retained the sample number for that group with the same number as the larger group; for undersampling, we resampled the larger group without replacement and retained the sample number for that group with the same number as the smaller group.

### Contaminant Identification

To control the levels of contamination, sterile water and a filter tip were used during extraction of DNA from the saliva samples. To identify and remove potential contaminants from environmental sources, we analyzed the reads of the taxa at the OTU level and excluded taxa if they appeared in only one sample or if the read counts were <2. Furthermore, we also analyzed bacteria with an average abundance of more than 1% in at least one sample.

## Results

### Demographics, Groups, and Oral Status (Caries and Black Stain)

We divided the population into two groups: the ECC and CF groups. The ECC group was divided into three subgroups: IDA ECC, NO IDA ECC and BS ECC. The CF group was also divided into three subgroups: BSCF, NBS CF, and IDA CF. Next, after random sampling in these populations, we included97 children with ECC (DMFS≥8) (NO IDA ECC, 80; IDA ECC, 13; and BS ECC, 4) and45 CF children (BSCF, 17; NO BSCF, 26; and IDA CF, 2). In the IDA population, the prevalence of caries was 81.5% (**Table S1-1**). Among them, the proportion of patients with IDA but without caries was low; after random sampling, we found only 2 children with IDA and without caries. Due to the small number of samples, no comparison between groups could be conducted. For the time being, this subgroup was removed. Similarly, there were only 4 children with ECC (DMFS≥8) who had pigmentation; therefore, we removed this subgroup. Finally, we included 93 people in the ECC group and 43 people in the CF group. Thirty-six children were excluded from this study because they did not meet the inclusion criteria; these children had systemic diseases; were under regular medication; refused to cooperate in dental examination, saliva collection, or routine blood examination; or were absent from school on the examination day.

The remaining 136 subjects included 43 CF children and 93 children with ECC. All subjects were examined by dental examination or saliva collection and blood routine examination. There was no significant difference in age among the different groups, including ECC vs. CF, NIDA ECC vs. IDA ECC and BSCF vs. NBSCE (all p value > 0.05). Moreover, there was no significant difference in sex among the three different groups (p > 0.05), except in IDA ECC vs. NIDA ECC (p = 0.04; **Table S2-1**, **S2-2**, **S2-3**).

In this study, we sequenced child saliva samples with Illumina MiSeq technology and obtained 11483411 raw sequences with an average length of 460 bp. After trimming, 11000078 reads of 16S rRNA with an average length of 464 bp were obtained. The species-level operational taxonomic units (OTUs) at 3% dissimilarity for each sample were filtered using the Uclust program (Edgar, 2010), and a total of 116734 OTUs were obtained from 136 samples, with 215 to 591 OTUs per sample. Altogether, 17 phyla, 27 classes, 51 orders, 92 families, 121 genera, and 433 bacterial species were represented by all the samples. There was no significant difference in salivary microbial profiles between the different sexes (p > 0.05).

### Composition and Structure of the Oral Salivary Microbiomes of 43 Carries-Free Children and 93 Children With Early Childhood Caries

The species-level OTUs and species richness and diversity estimates of each sample were calculated. There was no significant difference in the number of OTUs when the sample difference was 3%. In addition, comparing the rarefaction curves, the overall abundance of the two groups of OTUs was similar at a difference of 3%. We used Chao1 to measure the abundance of the salivary microbiota and Shannon and Simpson diversity indices to measure the diversity of the salivary microbiota. There were significant differences in the diversity indices between the CF children and children with ECC (p ≤ 0.005, p.shannon ≤ 0.001, p.simpson=0.0017 ≤ 0.005) (**Figure 2A**), and the salivary microbiota diversity in the CF group was lower than that in the ECC group.

Principal coordinate analysis (PCoA) showed that there was a significant difference between the ECC group and the CF group (**Figure 2B**).

**Figure 1:**
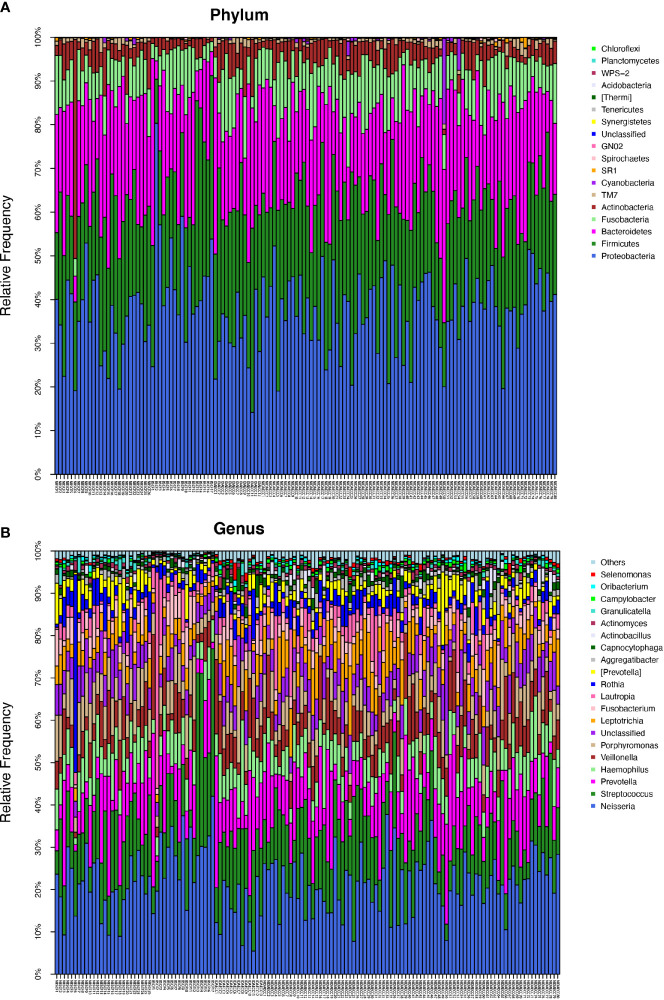
**(A)** The composition of phyla with ECC (including IDA ECC and NIDA ECC) and CF (including BSCF and NBSCF). Bar plot of percentage abundance at the phylum level in every sample. The horizontal axis is the name of each sample. They are ECC (including IDA ECC 1-13 and NIDA ECC 1-80) and CF (including BSCF 1-17 and NBSCF 1-26). **(B)** The composition of genera with ECC (including IDA ECC and NIDA ECC) and CF (including BSCF and NBSCF). Bar plot of percentage abundance at the genus level in every sample. The horizontal axis is the name of each sample. They are ECC (including IDA ECC 1-13 and NIDA ECC 1-80) and CF (including BSCF 1-17 and NBSCF 1-26).

The ECC group contained the following six dominant phyla (with a relative abundance larger than 1%) (Supplementary materials): Proteobacteria (36.9%), Firmicutes (25.3%), Bacteroidetes (21.6%), Fusobacteria (9.85%), and Actinobacteria (4.42%). The above bacteria accounted for 98.22% of the total composition (**Figure 1A**).

**Figure 2:**
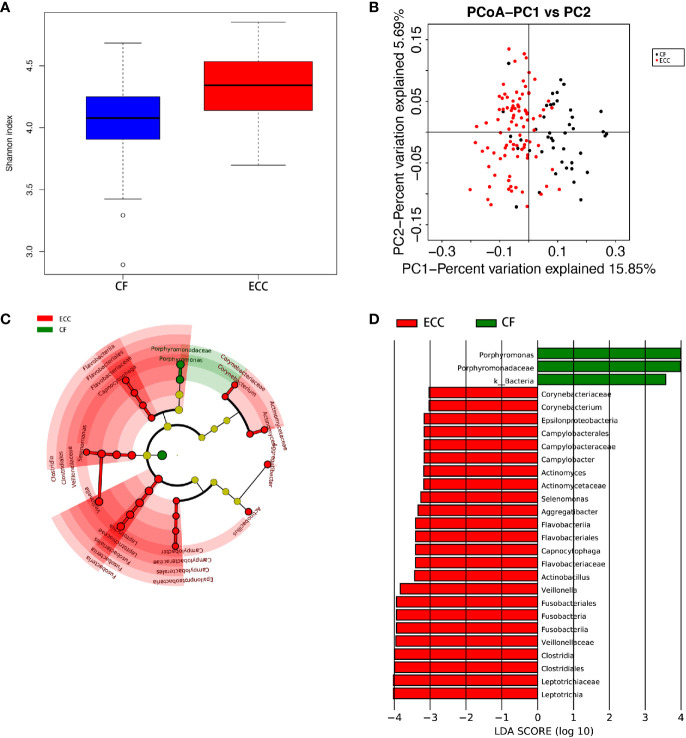
**(A)** Alpha diversity measurements using a Shannon index analysis indicated that ECC samples had higher genus diversity than CF samples. p.shannon ≤ 0.001. **(B)** Unweighted UnifracPCoA analysis. For each sample, the first two principal coordinates (PCo1 and PCo2) from the principal coordinate analysis of weighted UniFrac are plotted. The variance as calculated by PCoA is indicated in parentheses on the axes. ECC (black dots): saliva samples from ECC subjects. CF (red dots): saliva samples from CF subjects. **(C)** Cladogram of taxonomic biomarkers between the ECC and CF groups as identified by LEfSe. Green and red indicate data from the CF and ECC groups, respectively. **(D)** LDA score bar plot of taxonomic biomarkers between the ECC and CF groups as identified by LEfSe. Green represents the CF group. Genus of bacteria with high content. Red represents the ECC group. Genus of bacteria with high content.

The dominant bacterial phyla in the CF group were Proteobacteria (39.2%), Firmicutes (24.7%), Bacteroidetes (22.1%), Fusobacteria (8.03%), and Actinobacteria (4.83%), and these bacteria accounted for 98.91% of the total composition. There was no obvious difference in the above phyla between the two groups, but the abundances of Cyanobacteria, Spirochaetes, GN02, and Synergistetes were significantly different (p<0.05). (**Figure 1A**, **Figure 2C**) (Supplementary materials; for details, see **Table S3-1**).

There were 15 dominant genera in the ECC group, and the three genera with the highest abundances were Neisseria, Streptococcus and Prevotella. While there were 13 dominant genera in the CF group, the three genera with the highest abundances were the same as those in the ECC group. Moreover, there were 33 genera with significantly different relative abundances between the two groups (**Figure 1B**, **Figure 2C**) (Supplementary materials; for details, see **Table S4-1**).

**PICRUSt-based analysis** indicated obvious functional differences between the bacteria in the ECC and CF groups. The ECC microbiota tended to be rich in some KEGG pathways, such as protein digestion and absorption, phototransduction, and propanoate metabolism (Wilcox test), while KEGG pathway enrichment in CF bacteria was rare (Supplementary materials; for details, see **Table S5-1**).

### Oral Salivary Microbiome Diversity and Phylum- and Genus-Level Differences Between the Iron Deficiency Anemia Early Childhood Caries and NIDA Early Childhood Caries Groups

We calculated the species-level OTUs and species diversity and richness estimates for each sample. We did not observe a difference in the numbers of OTUs at 3% sample dissimilarity. We compared the rarefaction curves and observed that the two groups had a similar OTU richness at a 3% disparity level between the IDA ECC and NIDA ECC groups of children, which indicates that there was no significant difference in abundance (p>0.05). However, there were significant differences in the diversity indices between the IDA ECC and NO IDA ECC groups of children (p ≤ 0.05) (p.shannon=0.050 and p.simpson =0.024) (**Figure 4A**). The salivary microbiome diversity in the IDA ECC group was higher than that in the NIDA ECC group.

A comparison of beta diversity between the IDA ECC and NIDA ECC groups was conducted in this study. PCoA based on a weighted UniFrac matrix was used to identify differences in community structure and showed that the oral microbiota structure of the IDA ECC group differed from that of the NIDA ECC group. On the primary axes, the PCoA ordination did not reveal strong grouping of IDA ECC and NIDA ECC; however, a segregation trend for IDA ECC patients and NIDA ECC subjects was observed (**Figure 4B**).

PCoA between the two groups showed that there were differences in the microbiome between the two groups, but the differences were not very obvious. For analysis of the differences between the two groups at the phylum and genus levels, the Wilcoxon method was adopted, as shown in the table below (Supplementary materials; for details, see **Tables S3-2**, **4-2**).

**Figure 3:**
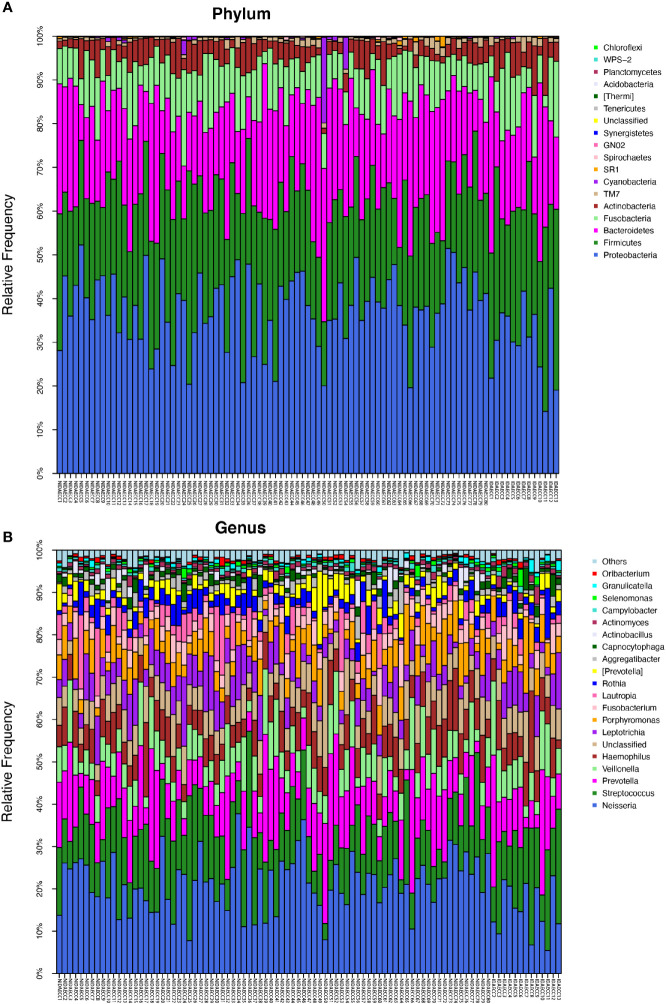
**(A)** The composition of phyla with NIDA ECC and IDA ECC. Bar plot of percentage abundance at the phylum level in every sample. The horizontal axis is the name of each sample. They are NIDA ECC1-80 and IDA ECC1-13. **(B)** The composition of genera with NIDA ECC and IDA ECC. Bar plot of percentage abundance at the genus level in every sample. The horizontal axis is the name of each sample. they are NIDA ECC1-80 and IDA ECC1-13.

**Figure 4:**
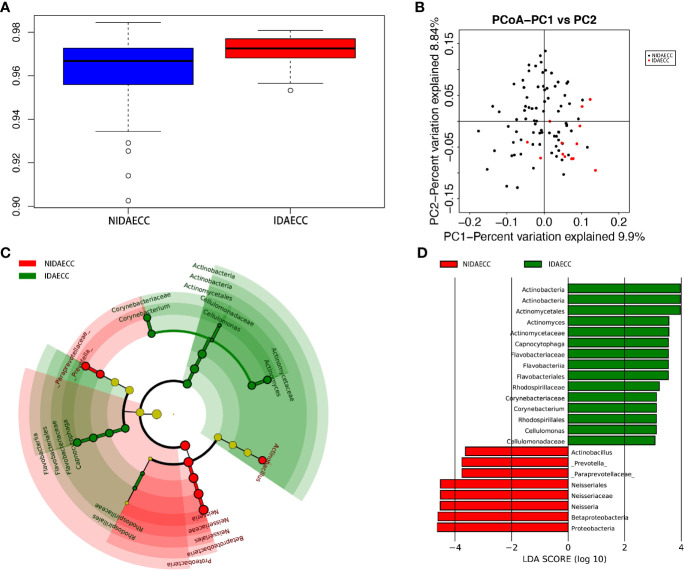
**(A)** Alpha diversity measurements using Simpson index analysis indicated that IDA ECCs had higher genus diversity than NIDA ECCs (p.simpson = 0.024 <0.05). **(B)** Unweighted UnifracPCoA analysis. For each sample, the first two principal coordinates (PCo1 and PCo2) from the principal coordinate analysis of weighted UniFrac are plotted. The variance as calculated by PCoA is indicated in parentheses on the axes. NIDA ECC (black dots): saliva samples from IDA-free ECC subjects. IDA ECC (red dots): saliva samples from IDA ECC subjects. **(C)** Cladogram of taxonomic biomarkers between the IDA ECC and NIDA ECC groups as identified by LEfSe. Green and red indicate data from the IDA ECC and NIDA ECC groups, respectively. **(D)** LDA score bar plot of taxonomic biomarkers between the IDA ECC and NIDA ECC groups as identified by LEfSe. Green represents the IDA ECC group. Genus of bacteria with high content. Red represents the NIDA ECC group. Genus of bacteria with high content.

The microbial compositions of the IDA ECC and NIDA ECC groups were compared and were found to be different. Seventeen phyla were identified in the NIDA ECC group, while 13 phyla were identified in the IDA ECC group. The dominant phyla in the IDA ECC group were Proteobacteria (30.2%), Firmicutes (29.3%), Bacteroidetes (22.2%), Fusobacteria (10.5%), Actinobacteria (6.1%), and TM7 (1.3%) (**Figure 3A**). The above bacteria accounted for 99.6% of the total composition. The dominant phyla in the NIDA ECC group were Proteobacteria (38%), Firmicutes (24.8%), Bacteroidetes (21.5%), Fusobacteria (9.8%), Actinobacteria (4.1%), and TM7 (0.92%); these bacteria accounted for 99.1% of the total composition. Among these phyla, there were significant differences in the relative abundances of Cyanobacteria and Proteobacteria (P = 0.043) between the IDA ECC group and the NIDA ECC group (adjusted p < 0.05) (**Figure 4C**) (Supplementary materials; for details, see **Table S3-2**).

In total, 112 genera were found in the NIDA ECC group, while 80 genera were found in the IDA ECC group. There were 15 dominant genera in both groups, and 13 of the 15 dominant genera were the same for the two groups. The three genera with the highest relative abundances in both groups were Streptococcus, Neisseria and Prevotella. Among all the genera, the relative abundances of four genera between the N IDA ECC and IDA ECC groups were significantly different, namely, Neisseria (21.75% vs. 15.04%, P = 0.046), Bacillus (0.0016% vs. 0.013%, P = 0.023), Moraxella (0.13% vs. 0.22%, P = 0.011), and Rhodococcus (0.0026% vs. 0.017%, P =0.011); in addition, there was a difference in the abundance of Actinomyces (0.8% vs. 1.56%, P=0.057) **(** **Figure 3B**, **Figure 4C**) (Supplementary materials; for details, see **Table S4-2**).

Consistent with previous results, **PICRUSt-based analysis** indicated obvious functional differences between the IDA and NO IDA groups, and the functional differences focused on various metabolism and biosynthesis processes (**Table S5–2**).

### Oral Salivary Microbiome Diversity and Phylum- and Genus-Level Differences Between the Black Stain Caries-Free and Nonblack Stain Caries-Free (Healthy Children) Groups

The average species richness (Chao1) of the samples within each subgroup was not different, but between the BSCF and NBS CF groups, there were significant differences in the diversity indices (p < 0.05) **(** **Figure 6A**), and the BSCF group had lower diversity.

**Figure 5:**
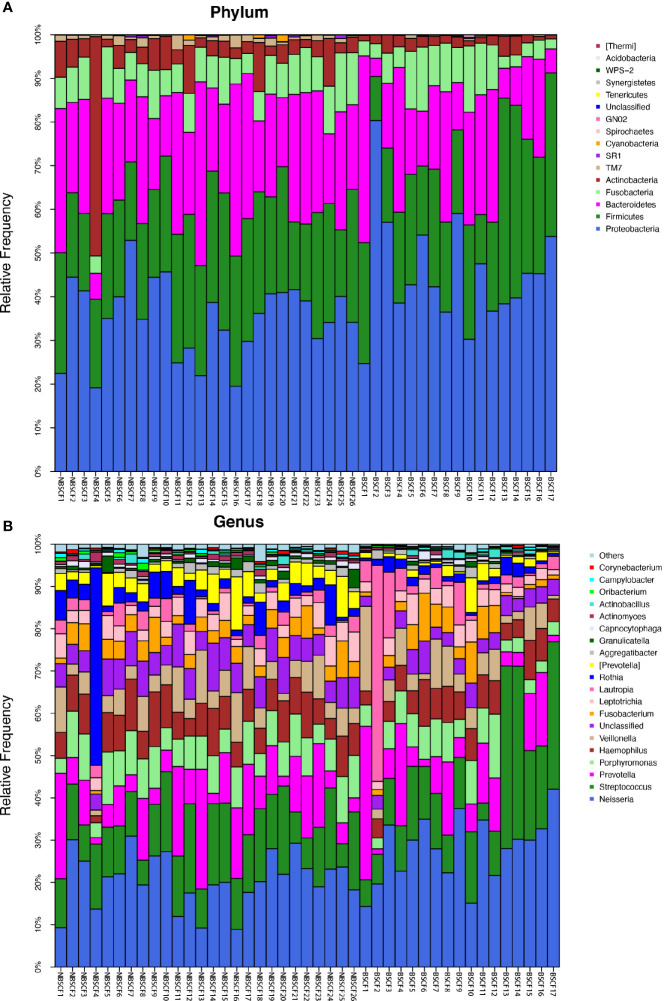
**(A)** The composition of phyla with NBSCF and BSCF. Bar plot of percentage abundance at the phylum level in every sample. The horizontal axis is the name of each sample. They are NBSCF1-26 and BSCF1-17. **(B)** The composition of genera with NBSCF and BSCF. Bar plot of percentage abundance at the genus level in every sample. The horizontal axis is the name of each sample. They are NBSCF1-26 and BSCF1-17.

**Figure 6:**
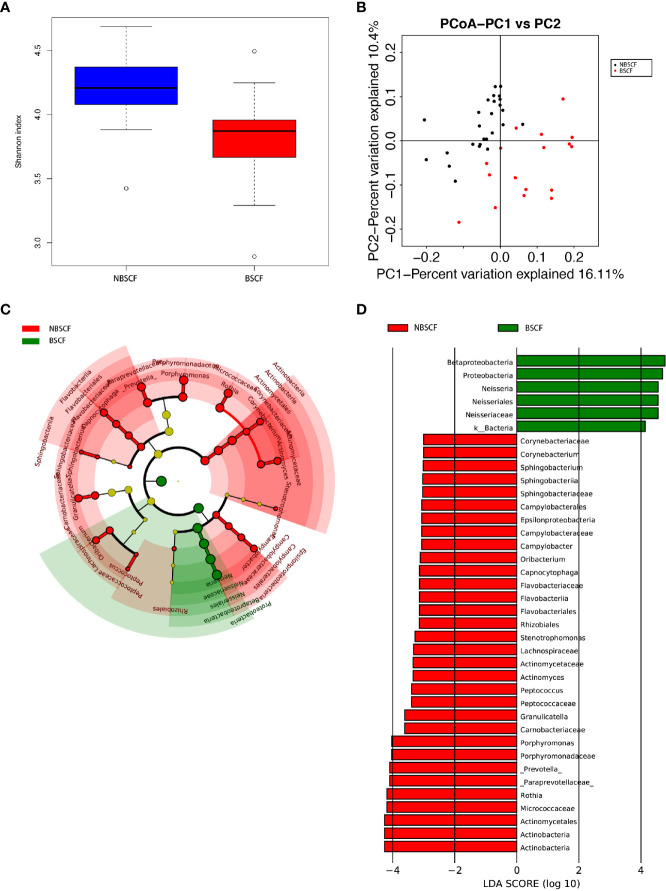
**(A)** Alpha diversity measurements using a Shannon index analysis indicated that NBSCF samples had higher genus diversity than BSCF samples. p.shannon = 0.00002 < 0.05. **(B)** Unweighted UnifracPCoA analysis. For each sample, the first two principal coordinates (PCo1 and PCo2) from the principal coordinate analysis of weighted UniFrac are plotted. The variance as calculated by PCoA is indicated in parentheses on the axes. NBSCF (black dots): saliva samples from NBSCF subjects. BSCF (red dots): saliva samples from BSCF subjects. **(C)** Cladogram of taxonomic biomarkers between the nonblack stain caries-free (NBSCF) and black stain caries-free (BSCF) groups as identified by LEfSe. Green and red indicate data from the BSCF and NBSCF groups, respectively. **(D)** LDA score bar plot of taxonomic biomarkers between nonblack stain caries-free (NBSCF) and black stain caries-free (BSCF) groups as identified by LEfSe. Green represents the BSCF group genus of bacteria with high content. Red represents the NBSCF group genus of bacteria with high content.

A comparison of beta diversity between the BSCF and NBSCF groups was conducted in this study. PCoA based on a weighted UniFrac matrix was used to identify differences in community structure and showed that the oral microbiota structure of the BSCF group differed from that of the NBSCF group. On the primary axes, the PCoA ordination did reveal strong grouping of BSCF and NBSCF, and BSCF and NBSCF showed a significant separation trend (**Figure 6B**). **Sixteen** phyla were identified in the NBS CF group, while 13 phyla were identified in the BSCF group. The dominant phyla were Proteobacteria (45.42%), Firmicutes (25.12%), Bacteroidetes (19.31%), Fusobacteria (7.47%) and Actinobacteria (2.26%) in the BSCF group. The dominant phyla in the NBS CF group were Proteobacteria (35.11%), Firmicutes (24.46%), Bacteroidetes (23.94%), Fusobacteria (8.41%), Actinobacteria (6.51%) and TM7 (1.15%)(**Figure 5A**). The relative abundances of six phyla were significantly different between the two groups (p < 0.05): Cyanobacteria, TM7, Actinobacteria, GN02, Proteobacteria and SR1. The phyla TM7, Cyanobacteria, Actinobacteria, GN02 and SR1 had significantly lower relative abundances in the NBS CF group than in the BSCF group (both p < 0.05), while the opposite was observed for Proteobacteria (**Figure 6C**) (Supplementary materials; for details, **Table S3-3**).

**Ninety-nine** genera were found in the NBS CF group, while 58 genera were found in the BSCF group. There was a significant difference in diversity between the two groups. There were 12 and 14 dominant genera in the BSCF and NBS CF groups, respectively. There were significant differences in 21 genera between the two groups (all adjusted p < 0.05). Among these genera, 21 had a significantly higher relative abundance in the NBS CF group than in the BSCF group (both adjusted p < 0.05). In contrast, the genus Neisseria showed the opposite trend, exhibiting greater abundance in the BSCF group (**Figure 5B**, **Figure 6C**) (Supplementary materials; for details, see **Table S4-3**).

PICRUSt-based analysis indicated obvious functional differences between the BSCF and NBS CF samples, and the functional differences were focused on various cellular signaling pathways and metabolic processes (**TableS5-3**).

### Potentially Dangerous Biomarkers Discovered by Comparison of Relative Abundances

Despite the overall similarity of microbial structure between the ECC group and the CF group, linear discriminant analysis (LDA) identied multiple differentially abundant taxa in each of the two groups; 24 taxa had high relative abundance in the ECC group, while 3 taxa had high relative abundance in the CF group (**Figure 2D**). At the genus level, 9 and 1 genera had higher relative abundances in the ECC group and the CF group, respectively.

LDA also identified 23 different taxa between the IDA ECC and NIDA ECC groups; 15 taxa had a higher relative abundance in the IDA ECC group, while 8 taxa had a higher relative abundance in the NO IDA ECC group (**Figure 4D**). At the genus level, 3 genera had higher abundances in the NO IDA ECC group, i.e., Actinobacillus, Neisseria and Prevotella, while there were 4 genera with higher abundances in the IDA ECC group, i.e., Actinomyces, Corynebacterium, Capnocytophaga, and Cellulomonas.

LDA also identified 38 different taxa between the **BSCF and NBS CF groups**. Thirty-two taxa had a higher relative abundance in the **NBS CF** group, while 6 taxa had a higher relative abundance in the **BSCF** group.(**Figure 6D**). At the genus level, only Neisseria had a higher relative abundance in the **BSCF** group, while12 genera had a higher relative abundance in the **NBS CF** group, i.e., Stenotrophomonas, Actinomyces, Corynebacterium, Rothia, Porphyromonas, Prevotella, Capnocytophaga, Sphingobacterium, Granulicatella, Oribacterium, Peptococcus, and Campylobacter. Interestingly, both Neisseria and Campylobacter belong to the phylum Proteobacteria.

Next, we established a random forest classifier using these biomarkers to predict disease outcomes. The random forest classifier achieved the best accuracy (93.35%) in predicting the outcome of caries using seven biomarkers. For the best classifier to predict the outcome of anemia and black stain, the included biomarkers were one and eight, respectively, and the accuracies were 94.62% and 95.23%, respectively. Considering the imbalance of the number of samples between groups, we also computed the accuracies by oversampling and undersampling. The accuracies of the three classifiers were 89.07%, 81.79% and 89.32% under sampling, respectively, while the accuracies of the classifiers were 96.57%, 99.19% and 95.90% for oversampling, respectively.

## Discussion

ECC was ranked as the 12th most prevalent disease, affecting approximately 560 million children worldwide in 2015 Global Burden of Disease Study (2016). The children experience extreme discomfort, pain, inability to eat, sleep disturbance, and further periapical periodontitis. Anemia is a global public health problem that affects a quarter of the world’s population and is concentrated in preschool children and women. The prevalence of anemia is estimated to be 47.4% for preschool children (McLean et al., 2009). Our previous study found that the caries incidence in children with anemia reach 81.5% (Gao et al., 2019a; Gao et al., 2019b). Studies have shown that children with low hemoglobin content are more susceptible to caries (Tang et al., 2013; Bansal et al., 2016), and young children with iron deficiency are more likely to suffer from ECC (Iranna Koppal et al., 2013). In this study, among the 1584 young children, 38 were diagnosed with anemia; the caries incidence, average number of caries, and the number of caries in the anemia group were significantly higher than those in the non-anemia group. However, the prevalence of caries in children with BS on teeth is much lower than that in the normal population (Garan et al., 2012; de Rezende et al., 2019). Our study further implies that Iron deficiency anemia (IDA) is a high-risk factor but extrinsic black stain on the tooth surface is a protective factor for caries.

The imbalance of micro-ecological status is the pathogenesis of dental caries. In this study, we sequenced child saliva samples with Illumina MiSeq technology and obtained raw sequences with an average length of 460 bp. Principal coordinate analysis showed that there was a significant difference of composition and structure of the oral salivary microbiomes between the ECC group and the CF group. In the ECC group, the three genera with the highest abundances were Neisseria, Streptococcus and Prevotella same as those in the CF group. Moreover, there were 33 genera with significantly different relative abundances between the two groups. Regarding diversity, different scholars have different views. Some scholars believe that increased alpha diversity is associated with health (Hurley et al., 2019); however, other studies report the opposite (Xu et al., 2014; Johansson et al., 2016). One study suggested that the Shannon index of subjects with caries is numerically greater than that of CF subjects, and the OTU number of subjects with caries is also higher than that of the CF group, which is consistent with the viewpoint of this paper (Jiang et al., 2016). Wang et al. found that the diversity of the caries group was slightly higher than that of the CF group. However, there was no statistically significant difference between the two groups (p > 0.05) (Wang et al., 2017). This result is similar to ours, but we observed significant differences between groups. These results suggest that the correlation of species richness with caries status is not the same in all populations, which may be caused by the environment.

In order to observe whether the children with IDA and extrinsic BS (the high-risk factors and protective factors of caries) affect oral microecological balance, we divided into two kind of subgroups: one is IDAECC group and NIDAECC group, another is BSCF group and NBSCF group to identify the differences in their flora and further identified the cariogenic flora and the protective flora that do not easily cause caries. Firstly, Oral salivary microbiome diversity and phylum- and genus-level appeared differences between the IDA ECC and NIDA ECC groups. 112 genera were found in the NIDA ECC group, while 80 genera were found in the IDA ECC group. The three genera with the highest relative abundances in both groups were Streptococcus, Neisseria and Prevotella. Among all the genera, Neisseria, Bacillus, Moraxella and Rhodococcus between the NIDA ECC and IDA ECC groups were significantly different. Secondly, Oral salivary microbiome diversity and phylum- and genus-level differences were analyzed between the BSCF and NBS CF group. There were significant differences in the diversity indices between two groups and the BSCF group had lower diversity. Only Neisseria exhibited high abundance in the BSCF, and the remaining genera exhibited high abundance in the NBS CF. Interestingly, the BSCF had the same trend as the NIDAECC, and the opposite trend was observed with IDA ECC. Thirdly, to predict ECC, IDA and BS, classifiers was established for the biomarkers screened in different groups, and classifiers have high accuracy and can predict the occurrence of the above diseases.

As one of the trace elements necessary for the growth of almost all living organisms, iron plays an important role in determining biological abundance and biodiversity in various ecosystems. Renke Wang’s studies have shown that iron in saliva can regulate the composition of oral salivary microbial communities (Wang et al., 2012). Tripodi D analyzed the microbes in the saliva and subgingival plaque of people with BS and found that actinomycetes are predominant in the pigment belt and saliva, and *S. mutans* and *Lactobacillus acidophilus* were present at relatively low levels. This is presumed to be a reason for the low rate of caries in the pigmentation group (Tripodi et al., 2016). Other studies have found that saliva in the pigmentation group contains more calcium, inorganic phosphate, etc., and the deposition of calcium and fluorine in the pigmentation zone reduces the dissolution of enamel, which makes caries formation difficult (SUN et al., 2013).In this study, we compare and analyze the oral flora of people with anemia and extrinsic BS to identify the bacterial markers related to caries and the protective flora of caries. Anemia and pigmentation were two closely related factors related to the formation of dental caries, the studies of which could help us to reveal the mechanism of caries formation playing by microbes. The point is this study showed salivary microbiome variation and selected biomarker can achieve good prediction results, suggesting their potential clinical implications although we could not explain variation mechanism.

We chose the saliva of children as samples because it is easily available and contains a large number of detectable oral microorganisms. These bacteria have potential applications as biomarkers and may cause clinical caries disease and BS tooth manifestations (Teng et al., 2015).The bacterial components in saliva can also represent the bacteria present on the oral mucosal surface (especially the tongue and buccal epithelium), so they are usually used to describe the oral microbiome (Hall et al., 2017; Sulyanto et al., 2019).It has been reported in the literature that the quantity of OTUs found in saliva is higher than that in gingival plaque and tongue plaque (Hall et al., 2017). However, the difference of microbiome variation between saliva and plaque needs further study.

## Data Availability Statement

The datasets presented in this study can be found in online repositories. The names of the repository/repositories and accession number(s) can be found below: NCBI BioProject [accession: PRJNA700472].

## Ethics Statement

The studies involving human participants were reviewed and approved by the Affiliated Hospital of Qingdao University. The patients’/participants’ legal guardian provided written informed consent to participate in this study.

## Author Contributions

All authors listed have made a substantial, direct, and intellectual contribution to the work, and approved it for publication.

## Funding

The authors are sincerely thankful to the National Natural Science Foundation of China (No.81670967), the Shandong Provincial Natural Science Foundation (no.2018GSF118167), and the Qingdao Science and Technology Plan Project (no.19-6-1-33-nsh) for providing financial support.

## Conflict of Interest

The authors declare that the research was conducted in the absence of any commercial or financial relationships that could be construed as a potential conflict of interest.
