# Supplementary material for: Salivary Microbiome Variation in Early Childhood Caries of Children 3–6 Years of Age and Its Association With Iron Deficiency Anemia and Extrinsic Black Stain
Source: Front Cell Infect Microbiol. 2021 Mar 23;11:628327. doi: 10.3389/fcimb.2021.628327 (PMC8044945; doi:10.3389/fcimb.2021.628327)
Supplement: Supplementary file 1 [file DataSheet_1.docx]

| Anemia or not | Number of people | No caries | caries | Caries prevalence（%） | dmft | | dmfs |
| --- | --- | --- | --- | --- | --- | --- | --- |
| No anemia | 1546 | 529 | 1017 | 65.8 | | 3.3 | 5.5 |
| anemia | 38 | 7 | 31 | 81.5 | | 5.8 | 10.5 |

**Table S1-1 Caries prevalence about** **Anemia and no Anemia in preschool children**

**TABLE S2-1 caries free and ECC**

|  |  | CARIES FREE | ECC |  |  |
| --- | --- | --- | --- | --- | --- |
| Age |  | 65.35$\pm$12.02 | 67.03$\pm9.62$ | t=－0.875 | P=0.383 |
| Gender | Boy  Girl | 27（33.8%）  16（28.6%） | 53（66.3%）  40（71.4%） | X^2^=0.409 | P=0.523 |
| Dmfs |  | 0 | 20.36$\pm9.36$ | － | － |
| Black stain | no  mild  moderate  serious  total | 26（21.8%）  8（100%）  5（100%）  4（100%）  43 | 93（78.2%）  0（0%）  0（0%）  0（0%）  93 | X^2^=42.020 | P=0.000 |
| HGB  MCV  MCH  MCHC |  | 134.37$\pm$9.57  83.71$\pm4.32$  28.41$\pm$0.80  322.69$\pm$7.49 | 133.49$\pm10.86$  82.85$\pm4.64$  28.39$\pm1.44$  320.08$\pm12.58$ | t=0.450  t=1.019  t=0.086  t=1.512 | P=0.653  P=0.310  P=0.931  P=0.133 |

**TABLE S2-2 ECC ( IDA ECC and NO IDA ECC)**

|  |  | IDA ECC | NO IDA ECC |  |  |
| --- | --- | --- | --- | --- | --- |
| Age |  | 63.23$\pm11.54$ | 67.65$\pm9.21$ | t=－1.548 | P=0.125 |
| Gender | Boys  Girls  Total | 4（7.5%）  9（22.5%）  13（14%） | 49（92.5%）  31（77.5%）  80（86.0%） | X^2^=4.239 | P=0.040 |
| Dmfs |  | 21.62$\pm$8.18 | 20.15$\pm9.57$ | t=0.521 | P=0.603 |
| Black stain |  | 0 | 0 | － | － |
| HGB  MCV  MCH  MCHC |  | 108.46$\pm1.27$  76.25$\pm1.83$  $26.35\pm0.94$  $297.39\pm8.50$ | 135.50$\pm8.79$  83.93$\pm4.02$  28.72$\pm1.22$  323.76$\pm8.66$ | t=－25.905  t=－11.314  t=－6.648  t=－10.215 | P=0.000  P=0.00  P=0.000  P=0.000 |

**TABLE S2-3 Caries free groups(NBSCF and BSCF)**

|  |  | NBSCF | BSCF |  |  |
| --- | --- | --- | --- | --- | --- |
| Age |  | 63.27$\pm11.7$ | 68.53$\pm12.14$ | t=－1.420 | P=0.163 |
| Gender | boys  girls | 16（59.3%）  10（62.5%） | 11（40.7%）  6（37.5%） | X^2^=0.044 | P=0.834 |
| Dmfs |  | 0 | 0 | － | － |
| Black stain | No  Mild  moderate  serious | 26  0  0  0 | 0  8  5  4 | X^2^=43.00 | P=0.000 |
| HGB  MCV  MCH  MCHC |  | 133.46$\pm$9.33  83.90$\pm4.36$  28.35$\pm$0.85  317.40$\pm$13.21 | 135.77$\pm10.04$  83.41$\pm4.37$  28.49$\pm0.72$  316.18$\pm14.55$ | t=－0.768  t=0.359  t=-0.537  t=-0.088 | P=0.447  P=0.722  P=0.594  P=0.930 |

**TABLE S3-1 The different phylum in CARIES-FREE and ECC**

| taxa | CF  n | ECC  n | wilcox.pvalue | FDR |
| --- | --- | --- | --- | --- |
| k__Bacteria;p__Cyanobacteria | 113 | 490.4301 | 2.40E-07 | 4.31E-06 |
| k__Bacteria;p__Spirochaetes | 29.34884 | 84.90323 | 1.26E-06 | 1.14E-05 |
| k__Bacteria;p__GN02 | 15.18605 | 37.83871 | 6.34E-05 | 0.000381 |
| k__Bacteria;p__Synergistetes | 2.232558 | 9.096774 | 0.003389 | 0.015249 |

**TABLE S3-2 The different phylum in IDA ECC AND NIDA ECC**

| taxa | NIDAECC  n | IDAECC n | wilcox.pvalue | FDR |
| --- | --- | --- | --- | --- |
| k__Bacteria;p__Cyanobacteria | 556.6375 | 83 | 0.000193 | 0.00347 |
| k__Bacteria;p__Proteobacteria | 38000 | 30279.69 | 0.004807 | 0.043266 |

**TABLE S3-3 The different phylum in BSCF and NBSCF**

| taxa | NBSCF  n | BSCF  n | wilcox.pvalue | FDR |
| --- | --- | --- | --- | --- |
| k__Bacteria;p__TM7 | 1151 | 238.1765 | 4.89E-07 | 7.82E-06 |
| k__Bacteria;p__Cyanobacteria | 173.8462 | 19.94118 | 0.000195 | 0.001556 |
| k__Bacteria;p__Actinobacteria | 6517.538 | 2261 | 0.000309 | 0.00165 |
| k__Bacteria;p__GN02 | 23.15385 | 3 | 0.000632 | 0.002528 |
| k__Bacteria;p__Proteobacteria | 35113.27 | 45442.12 | 0.006085 | 0.019473 |
| k__Bacteria;p__SR1 | 168.1538 | 74.05882 | 0.012495 | 0.03332 |

**TABLES4-1**  **THE DIFFERENCE GENUS IN CF AND ECC GROUPS**

| taxa | CF.mean | ECC.mean | wilcox.pvalue | FDR |
| --- | --- | --- | --- | --- |
| k__Bacteria;p__Firmicutes;c__Bacilli;o__Lactobacillales;f__Lactobacillaceae;g__Lactobacillus | 1.55814 | 30.01075 | 9.29E-13 | 1.12E-10 |
| k__Bacteria;p__Firmicutes;c__Clostridia;o__Clostridiales;f__Veillonellaceae;g__Selenomonas | 175.2326 | 532.5484 | 2.57E-11 | 1.56E-09 |
| k__Bacteria;p__Firmicutes;c__Clostridia;o__Clostridiales;f__Veillonellaceae;g__Dialister | 8.023256 | 41.2043 | 8.94E-11 | 3.61E-09 |
| k__Bacteria;p__Actinobacteria;c__Coriobacteriia;o__Coriobacteriales;f__Coriobacteriaceae;g__Atopobium | 19.62791 | 66.32258 | 1.90E-10 | 5.76E-09 |
| k__Bacteria;p__Firmicutes;c__Clostridia;o__Clostridiales;f__Veillonellaceae;g__Megasphaera | 51.16279 | 147.9355 | 2.32E-08 | 5.62E-07 |
| k__Bacteria;p__Bacteroidetes;c__Bacteroidia;o__Bacteroidales;f__Porphyromonadaceae;g__Tannerella | 21.16279 | 57.63441 | 3.27E-08 | 6.60E-07 |
| k__Bacteria;p__Proteobacteria;c__Epsilonproteobacteria;o__Campylobacterales;f__Campylobacteraceae;g__Campylobacter | 345.4884 | 645.7742 | 9.06E-08 | 1.57E-06 |
| k__Bacteria;p__Actinobacteria;c__Actinobacteria;o__Bifidobacteriales;f__Bifidobacteriaceae;g__Scardovia | 0.534884 | 7.258065 | 1.26E-07 | 1.91E-06 |
| k__Bacteria;p__Firmicutes;c__Clostridia;o__Clostridiales;f__Veillonellaceae;g__Schwartzia | 4.023256 | 16.24731 | 3.40E-07 | 4.57E-06 |
| k__Bacteria;p__Firmicutes;c__Clostridia;o__Clostridiales;f__Lachnospiraceae;g__Moryella | 20 | 51.52688 | 8.03E-07 | 9.72E-06 |
| k__Bacteria;p__Firmicutes;c__Clostridia;o__Clostridiales;f__[Mogibacteriaceae];g__Anaerovorax | 21.23256 | 6.677419 | 1.09E-06 | 1.20E-05 |
| k__Bacteria;p__Spirochaetes;c__Spirochaetes;o__Spirochaetales;f__Spirochaetaceae;g__Treponema | 29.34884 | 84.90323 | 1.26E-06 | 1.27E-05 |
| k__Bacteria;p__Proteobacteria;c__Betaproteobacteria;o__Neisseriales;f__Neisseriaceae;g__Kingella | 19 | 45.39785 | 1.17E-05 | 0.000109 |
| k__Bacteria;p__Firmicutes;c__Clostridia;o__Clostridiales;f__Lachnospiraceae;g__Shuttleworthia | 0.488372 | 5.752688 | 1.54E-05 | 0.000133 |
| k__Bacteria;p__Fusobacteria;c__Fusobacteriia;o__Fusobacteriales;f__Leptotrichiaceae;g__Leptotrichia | 3840.512 | 6056.204 | 5.37E-05 | 0.000433 |
| k__Bacteria;p__Bacteroidetes;c__Bacteroidia;o__Bacteroidales;f__Porphyromonadaceae;g__Paludibacter | 12.39535 | 24.04301 | 9.37E-05 | 0.000709 |
| k__Bacteria;p__Proteobacteria;c__Gammaproteobacteria;o__Cardiobacteriales;f__Cardiobacteriaceae;g__Cardiobacterium | 33.51163 | 61.58065 | 0.000107 | 0.000764 |
| k__Bacteria;p__Actinobacteria;c__Actinobacteria;o__Actinomycetales;f__Corynebacteriaceae;g__Corynebacterium | 245.1163 | 441.1935 | 0.000186 | 0.001206 |
| k__Bacteria;p__Actinobacteria;c__Actinobacteria;o__Bifidobacteriales;f__Bifidobacteriaceae;g__Bifidobacterium | 0.395349 | 4.290323 | 0.000189 | 0.001206 |
| k__Bacteria;p__Bacteroidetes;c__Flavobacteriia;o__Flavobacteriales;f__Flavobacteriaceae;g__Capnocytophaga | 701.186 | 1237.699 | 0.00074 | 0.004476 |
| k__Bacteria;p__Bacteroidetes;c__Bacteroidia;o__Bacteroidales;f__Porphyromonadaceae;g__Porphyromonas | 7544.558 | 5794.785 | 0.000847 | 0.004881 |
| k__Bacteria;p__Firmicutes;c__Erysipelotrichi;o__Erysipelotrichales;f__Erysipelotrichaceae;g__Bulleidia | 20.86047 | 35.3871 | 0.001212 | 0.006474 |
| k__Bacteria;p__Firmicutes;c__Bacilli;o__Lactobacillales;f__Aerococcaceae;g__Abiotrophia | 124.5814 | 214.871 | 0.001231 | 0.006474 |
| k__Bacteria;p__Proteobacteria;c__Betaproteobacteria;o__Neisseriales;f__Neisseriaceae;g__Eikenella | 119.8372 | 242.9462 | 0.001728 | 0.008711 |
| k__Bacteria;p__Firmicutes;c__Clostridia;o__Clostridiales;f__Eubacteriaceae;g__Pseudoramibacter_Eubacterium | 0 | 4.752688 | 0.002114 | 0.010208 |
| k__Bacteria;p__Firmicutes;c__Clostridia;o__Clostridiales;f__Peptostreptococcaceae;g__Filifactor | 3.674419 | 13.05376 | 0.002193 | 0.010208 |
| k__Bacteria;p__Actinobacteria;c__Actinobacteria;o__Actinomycetales;f__Actinomycetaceae;g__Actinomyces | 651.1395 | 907.9355 | 0.002937 | 0.013164 |
| k__Bacteria;p__Synergistetes;c__Synergistia;o__Synergistales;f__Dethiosulfovibrionaceae;g__TG5 | 2.232558 | 8.83871 | 0.003447 | 0.014898 |
| k__Bacteria;p__Proteobacteria;c__Gammaproteobacteria;o__Pasteurellales;f__Pasteurellaceae;g__Aggregatibacter | 1016.581 | 1530.247 | 0.005243 | 0.021877 |
| k__Bacteria;p__Proteobacteria;c__Gammaproteobacteria;o__Pasteurellales;f__Pasteurellaceae;g__Actinobacillus | 529 | 1077.484 | 0.009014 | 0.036358 |
| k__Bacteria;p__Proteobacteria;c__Alphaproteobacteria;o__Rhizobiales;f__Methylocystaceae;g__Methylopila | 0.302326 | 0 | 0.010559 | 0.041214 |
| k__Bacteria;p__Firmicutes;c__Clostridia;o__Clostridiales;f__Peptococcaceae;g__Peptococcus | 1.186047 | 3.473118 | 0.01259 | 0.047607 |
| k__Bacteria;p__Firmicutes;c__Clostridia;o__Clostridiales;f__Lachnospiraceae;g__Butyrivibrio | 38.4186 | 41.58065 | 0.013392 | 0.049104 |
| k__Bacteria;p__Firmicutes;c__Clostridia;o__Clostridiales;f__Veillonellaceae;g__Veillonella | 6039.628 | 7372.86 | 0.014378 | 0.051167 |

**TABLES4-2 THE DIFFERENCE GENUS IN NIDA ECC AND IDA ECC GROUPS**

| taxa | NIDA ECC.mean | IDA ECC.mean | wilcox.pvalue | FDR |
| --- | --- | --- | --- | --- |
| k__Bacteria;p__Actinobacteria;c__Actinobacteria;o__Actinomycetales;f__Nocardiaceae;g__Rhodococcus | 2.675 | 17.38462 | 0.000199 | 0.011699 |
| k__Bacteria;p__Proteobacteria;c__Gammaproteobacteria;o__Pseudomonadales;f__Moraxellaceae;g__Moraxella | 131.4 | 219.8462 | 0.000205 | 0.011699 |
| k__Bacteria;p__Firmicutes;c__Bacilli;o__Bacillales;f__Planococcaceae;g__Bacillus | 1.6 | 12.76923 | 0.000631 | 0.023972 |
| k__Bacteria;p__Proteobacteria;c__Betaproteobacteria;o__Neisseriales;f__Neisseriaceae;g__Neisseria | 21755.49 | 15040 | 0.001621 | 0.046208 |
| k__Bacteria;p__Actinobacteria;c__Actinobacteria;o__Actinomycetales;f__Actinomycetaceae;g__Actinomyces | 801.025 | 1565.846 | 0.002535 | 0.057801 |

**TABLES4-3 THE DIFFERENCE GENUS IN NBSCF AND BSCF GROUPS**

| taxa | NBSCF.mean | BSCF.mean | wilcox.pvalue | FDR |
| --- | --- | --- | --- | --- |
| Unclassified | 6678.962 | 4038.059 | 3.64E-06 | 0.000371 |
| k__Bacteria;p__Firmicutes;c__Clostridia;o__Clostridiales;f__Lachnospiraceae;g__Catonella | 146.2692 | 36.23529 | 2.02E-05 | 0.001033 |
| k__Bacteria;p__Firmicutes;c__Bacilli;o__Lactobacillales;f__Carnobacteriaceae;g__Granulicatella | 1049.577 | 305.5294 | 3.85E-05 | 0.001122 |
| k__Bacteria;p__Bacteroidetes;c__Bacteroidia;o__Bacteroidales;f__[Paraprevotellaceae];g__[Prevotella] | 4263.769 | 1788.706 | 4.40E-05 | 0.001122 |
| k__Bacteria;p__Actinobacteria;c__Actinobacteria;o__Actinomycetales;f__Actinomycetaceae;g__Actinomyces | 830.6538 | 376.5882 | 7.40E-05 | 0.001509 |
| k__Bacteria;p__Proteobacteria;c__Gammaproteobacteria;o__Pseudomonadales;f__Moraxellaceae;g__Acinetobacter | 16.65385 | 0.411765 | 9.01E-05 | 0.001532 |
| k__Bacteria;p__Actinobacteria;c__Actinobacteria;o__Actinomycetales;f__Corynebacteriaceae;g__Corynebacterium | 322.0385 | 127.4706 | 0.000152 | 0.002211 |
| k__Bacteria;p__Actinobacteria;c__Actinobacteria;o__Actinomycetales;f__Streptomycetaceae;g__Streptomyces | 39.61538 | 3.176471 | 0.000191 | 0.002435 |
| k__Bacteria;p__Proteobacteria;c__Betaproteobacteria;o__Neisseriales;f__Neisseriaceae;g__Kingella | 27.15385 | 6.529412 | 0.000397 | 0.004498 |
| k__Bacteria;p__Firmicutes;c__Clostridia;o__Clostridiales;f__Lachnospiraceae;g__Butyrivibrio | 57.15385 | 9.764706 | 0.000587 | 0.00599 |
| k__Bacteria;p__Actinobacteria;c__Coriobacteriia;o__Coriobacteriales;f__Coriobacteriaceae;g__Atopobium | 28.26923 | 6.411765 | 0.000947 | 0.008785 |
| k__Bacteria;p__Firmicutes;c__Clostridia;o__Clostridiales;f__Lachnospiraceae;g__Oribacterium | 461.7692 | 206.7647 | 0.001041 | 0.008847 |
| k__Bacteria;p__Proteobacteria;c__Gammaproteobacteria;o__Cardiobacteriales;f__Cardiobacteriaceae;g__Cardiobacterium | 45.88462 | 14.58824 | 0.001225 | 0.009611 |
| k__Bacteria;p__Actinobacteria;c__Actinobacteria;o__Actinomycetales;f__Nocardiaceae;g__Rhodococcus | 8.730769 | 0.470588 | 0.001379 | 0.010049 |
| k__Bacteria;p__Firmicutes;c__Erysipelotrichi;o__Erysipelotrichales;f__Erysipelotrichaceae;g__Bulleidia | 29.23077 | 8.058824 | 0.001737 | 0.011812 |
| k__Bacteria;p__Proteobacteria;c__Epsilonproteobacteria;o__Campylobacterales;f__Campylobacteraceae;g__Campylobacter | 435.0769 | 208.4706 | 0.002156 | 0.013744 |
| k__Bacteria;p__Proteobacteria;c__Betaproteobacteria;o__Neisseriales;f__Neisseriaceae;g__Neisseria | 20653.31 | 28083.71 | 0.002347 | 0.014083 |
| k__Bacteria;p__Actinobacteria;c__Actinobacteria;o__Actinomycetales;f__Micrococcaceae;g__Rothia | 5201.154 | 1710.882 | 0.004742 | 0.02687 |
| k__Bacteria;p__Firmicutes;c__Bacilli;o__Lactobacillales;f__Aerococcaceae;g__Abiotrophia | 138.3077 | 103.5882 | 0.00582 | 0.031243 |
| k__Bacteria;p__Firmicutes;c__Bacilli;o__Bacillales;f__Planococcaceae;g__Bacillus | 13.73077 | 0.705882 | 0.006785 | 0.034604 |
| k__Bacteria;p__Bacteroidetes;c__Bacteroidia;o__Bacteroidales;f__Porphyromonadaceae;g__Tannerella | 29.15385 | 8.941176 | 0.007635 | 0.03653 |
| k__Bacteria;p__Firmicutes;c__Clostridia;o__Clostridiales;f__Peptococcaceae;g__Peptococcus | 1.961538 | 0 | 0.007879 | 0.03653 |

**TABLE S5-1 ECC and CARIES FREE(CF) Oral saliva microbiome PICRUSt-based analysis**

| KEGGpathway | CARIES FREE.mean | CARIES.mean | wilcox.pvalue | FDR |
| --- | --- | --- | --- | --- |
| Photosynthesis - antenna proteins | 18.86047 | 73.65591 | 2.97E-07 | 3.80E-05 |
| 1,1,1-Trichloro-2,2-bis(4-chlorophenyl)ethane (DDT) degradation | 2.302326 | 4.365591 | 4.23E-07 | 3.80E-05 |
| Steroid biosynthesis | 2.627907 | 9 | 2.71E-07 | 3.80E-05 |
| Calcium signaling pathway | 1.023256 | 4.27957 | 5.41E-06 | 0.000364 |
| Bacterial chemotaxis | 849.3488 | 1011.849 | 1.65E-05 | 0.000886 |
| Phenylpropanoid biosynthesis | 259.9767 | 309.1828 | 8.36E-05 | 0.003748 |
| Photosynthesis proteins | 3918.86 | 4255.817 | 9.77E-05 | 0.003754 |
| Ascorbate and aldarate metabolism | 807 | 906.914 | 0.000116 | 0.003906 |
| Vitamin B6 metabolism | 2196.209 | 2140.796 | 0.000144 | 0.004317 |
| Pentose and glucuronate interconversions | 2032.372 | 2139.753 | 0.000181 | 0.004426 |
| Biosynthesis of type II polyketide products | 0.534884 | 1.150538 | 0.000178 | 0.004426 |
| Biosynthesis of ansamycins | 673.1628 | 726.8602 | 0.000261 | 0.005861 |
| Photosynthesis | 3823.558 | 4103.86 | 0.000372 | 0.007696 |
| Type II diabetes mellitus | 474.6744 | 494.9032 | 0.000537 | 0.010313 |
| Flavonoid biosynthesis | 47.44186 | 68.11828 | 0.000816 | 0.01464 |
| Folate biosynthesis | 5418.581 | 5305.387 | 0.000891 | 0.014976 |
| DNA repair and recombination proteins | 32350.51 | 31987.98 | 0.001027 | 0.016246 |
| Sporulation | 297.8837 | 352.9032 | 0.001133 | 0.016936 |
| Bladder cancer | 5.162791 | 7.301075 | 0.001206 | 0.01708 |
| beta-Lactam resistance | 76.69767 | 95.47312 | 0.001388 | 0.018671 |
| Secondary bile acid biosynthesis | 6.44186 | 4.193548 | 0.001458 | 0.018671 |
| Transcription related proteins | 19.95349 | 26.69892 | 0.001573 | 0.019233 |
| DNA replication proteins | 13477.28 | 13239.11 | 0.001947 | 0.022479 |
| Primary immunodeficiency | 653.3953 | 631.3011 | 0.002256 | 0.022479 |
| Various types of N-glycan biosynthesis | 11.86047 | 15.97849 | 0.002226 | 0.022479 |
| Primary bile acid biosynthesis | 7 | 4.666667 | 0.002159 | 0.022479 |
| Germination | 4.581395 | 5.397849 | 0.002178 | 0.022479 |
| Flagellar assembly | 820.186 | 917.3978 | 0.002368 | 0.022746 |
| Bile secretion | 0.465116 | 0.892473 | 0.002751 | 0.025514 |
| Meiosis - yeast | 67.83721 | 82.96774 | 0.003024 | 0.026243 |
| D-Arginine and D-ornithine metabolism | 45.60465 | 52.64516 | 0.002951 | 0.026243 |
| Cell motility and secretion | 2114.884 | 2214.645 | 0.004305 | 0.036188 |
| ABC transporters | 28257.56 | 29212.67 | 0.004806 | 0.038023 |
| Peptidoglycan biosynthesis | 9622.465 | 9515.022 | 0.004947 | 0.038023 |
| Stilbenoid, diarylheptanoid and gingerol biosynthesis | 70.93023 | 82.17204 | 0.0048 | 0.038023 |
| Basal transcription factors | 14.02326 | 19.08602 | 0.005282 | 0.039469 |

**TABLE S5-2**

**The iron deficiency anemia (IDA) ECC and** **NO iron deficiency anemia (IDA) ECC Oral saliva microbiome** **PICRUSt-based analysis**

| KEGGpathway | NIDA.mean | IDA.mean | wilcox.pvalue | FDR |
| --- | --- | --- | --- | --- |
| Oxidative phosphorylation | 12892.73 | 12145.23 | 0.001392 | 0.027051 |
| Amino sugar and nucleotide sugar metabolism | 12026.25 | 12835.54 | 0.00124 | 0.027051 |
| Bacterial secretion system | 7979.313 | 7643.846 | 0.001147 | 0.027051 |
| Pores ion channels | 7921.625 | 6958.846 | 0.000942 | 0.027051 |
| Selenocompound metabolism | 3733.963 | 3880.846 | 0.00082 | 0.027051 |
| Taurine and hypotaurine metabolism | 1389.25 | 1330.308 | 0.001146 | 0.027051 |
| Sphingolipid metabolism | 776.9625 | 939.3077 | 0.000483 | 0.027051 |
| Glycan biosynthesis and metabolism | 959.3875 | 804.6923 | 0.001418 | 0.027051 |
| Carotenoid biosynthesis | 209 | 136.0769 | 0.000376 | 0.027051 |
| Photosynthesis - antenna proteins | 83.3125 | 14.23077 | 0.000223 | 0.027051 |
| Bladder cancer | 6.3625 | 13.07692 | 0.000549 | 0.027051 |
| Endocytosis | 0.525 | 1.076923 | 0.000893 | 0.027051 |
| Fc gamma R-mediated phagocytosis | 0.525 | 1.076923 | 0.000893 | 0.027051 |
| GnRH signaling pathway | 0.525 | 1.076923 | 0.000893 | 0.027051 |
| Calcium signaling pathway | 4.85 | 0.769231 | 0.001869 | 0.033268 |
| Glutathione metabolism | 3694.525 | 3489.538 | 0.002069 | 0.033693 |
| Vibrio cholerae pathogenic cycle | 909.075 | 854.9231 | 0.002145 | 0.033693 |
| Lipid metabolism | 1470.85 | 1376.077 | 0.002488 | 0.036576 |
| Basal transcription factors | 17.6375 | 28 | 0.002603 | 0.036576 |
| Galactose metabolism | 4869.338 | 5486.692 | 0.003095 | 0.04001 |
| Proteasome | 243.2375 | 287.7692 | 0.003147 | 0.04001 |
| Parkinson's disease | 519.325 | 389.1538 | 0.003444 | 0.040681 |
| Ethylbenzene degradation | 204.8875 | 240.6923 | 0.003504 | 0.040681 |
| Secretion system | 16116.99 | 15185.77 | 0.006313 | 0.041193 |
| Aminoacyl-tRNA biosynthesis | 13719.55 | 13990.54 | 0.005327 | 0.041193 |
| Arginine and proline metabolism | 9895.113 | 9475.846 | 0.00698 | 0.041193 |
| Lipid biosynthesis proteins | 7208.388 | 6952.615 | 0.004257 | 0.041193 |
| Fructose and mannose metabolism | 5785.613 | 6359.538 | 0.006313 | 0.041193 |
| Bacterial motility proteins | 6157.388 | 4966.846 | 0.007097 | 0.041193 |
| Glyoxylate and dicarboxylate metabolism | 4444.663 | 4175.154 | 0.005238 | 0.041193 |
| Inorganic ion transport and metabolism | 3369.813 | 3006.462 | 0.006002 | 0.041193 |
| Valine, leucine and isoleucine degradation | 2586.213 | 2697.385 | 0.004257 | 0.041193 |
| Other transporters | 2704.338 | 2512.231 | 0.005801 | 0.041193 |
| Toluene degradation | 1740.538 | 1621.385 | 0.004485 | 0.041193 |
| Phenylalanine metabolism | 1550.5 | 1411.538 | 0.006527 | 0.041193 |
| Plant-pathogen interaction | 1252.525 | 1202.923 | 0.006743 | 0.041193 |
| Alzheimer's disease | 1201.638 | 1058.923 | 0.004724 | 0.041193 |
| Drug metabolism - cytochrome P450 | 1021 | 817.8462 | 0.004485 | 0.041193 |
| Metabolism of xenobiotics by cytochrome P450 | 1020.788 | 816.8462 | 0.004039 | 0.041193 |
| Retinol metabolism | 825.5125 | 694 | 0.005606 | 0.041193 |
| Biosynthesis and biodegradation of secondary metabolites | 542.975 | 489.2308 | 0.006859 | 0.041193 |
| Cardiac muscle contraction | 518.075 | 388.7692 | 0.003831 | 0.041193 |
| Mineral absorption | 301.45 | 269.9231 | 0.004971 | 0.041193 |
| Amyotrophic lateral sclerosis (ALS) | 273.6875 | 237.9231 | 0.006415 | 0.041193 |
| Penicillin and cephalosporin biosynthesis | 155.75 | 189.8462 | 0.006097 | 0.041193 |
| Steroid biosynthesis | 10.0625 | 2.461538 | 0.006225 | 0.041193 |
| D-Alanine metabolism | 1280.575 | 1353.154 | 0.007832 | 0.044494 |
| Renin-angiotensin system | 0.0125 | 0.153846 | 0.008225 | 0.04575 |
| Renal cell carcinoma | 275.2125 | 250 | 0.00877 | 0.047787 |
| Limonene and pinene degradation | 537.425 | 605.1538 | 0.009073 | 0.048449 |

**TABLE S5-3**

**The black stain caries free and** **no black stain caries free (health children) Oral saliva microbiome PICRUSt-based analysis**

| KEGGpathway | NBS.mean | BS.mean | wilcox.pvalue | FDR |
| --- | --- | --- | --- | --- |
| Basal transcription factors | 19.53846 | 5.588235 | 3.42E-07 | 9.20E-05 |
| Various types of N-glycan biosynthesis | 16.69231 | 4.470588 | 1.11E-06 | 0.00015 |
| Type I diabetes mellitus | 569.4231 | 522.6471 | 1.91E-05 | 0.001713 |
| Streptomycin biosynthesis | 3181.385 | 3299.529 | 5.13E-05 | 0.001972 |
| Pentose and glucuronate interconversions | 2110.308 | 1913.176 | 3.94E-05 | 0.001972 |
| Stilbenoid, diarylheptanoid and gingerol biosynthesis | 88.03846 | 44.76471 | 4.82E-05 | 0.001972 |
| Germination | 6.192308 | 2.117647 | 3.49E-05 | 0.001972 |
| Chagas disease (American trypanosomiasis) | 43 | 15.64706 | 0.000105 | 0.003127 |
| African trypanosomiasis | 42.84615 | 15.64706 | 0.000105 | 0.003127 |
| Sulfur metabolism | 3141.577 | 3400.118 | 0.000122 | 0.003268 |
| Bladder cancer | 6.576923 | 3 | 0.000134 | 0.003268 |
| Steroid biosynthesis | 4.115385 | 0.352941 | 0.000163 | 0.003654 |
| Photosynthesis - antenna proteins | 29.03846 | 3.294118 | 0.000221 | 0.004569 |
| Sulfur relay system | 2722.038 | 2553.353 | 0.000301 | 0.005782 |
| Fructose and mannose metabolism | 5991.115 | 5199.647 | 0.000364 | 0.006415 |
| Taurine and hypotaurine metabolism | 1384.308 | 1470.588 | 0.000382 | 0.006415 |
| Pores ion channels | 7663.846 | 8658.588 | 0.00048 | 0.007171 |
| Proteasome | 281.7692 | 189.5882 | 0.00046 | 0.007171 |
| 1,1,1-Trichloro-2,2-bis(4-chlorophenyl)ethane (DDT) degradation | 3.038462 | 1.176471 | 0.000621 | 0.00879 |
| Naphthalene degradation | 1605.423 | 1781.235 | 0.000797 | 0.009319 |
| Retinol metabolism | 761 | 943.1176 | 0.000731 | 0.009319 |
| Parkinson's disease | 471.6154 | 641.4706 | 0.000763 | 0.009319 |
| Cardiac muscle contraction | 470.6538 | 641.4118 | 0.000762 | 0.009319 |
| Phosphonate and phosphinate metabolism | 550.8846 | 636.9412 | 0.000953 | 0.010679 |
| Drug metabolism - cytochrome P450 | 931 | 1189.765 | 0.001096 | 0.01179 |
| N-Glycan biosynthesis | 262.5 | 172.6471 | 0.001293 | 0.013378 |
| Inorganic ion transport and metabolism | 3250.615 | 3717.235 | 0.001412 | 0.014067 |
| Function unknown | 18609.27 | 19907.76 | 0.001469 | 0.014109 |
| Metabolism of xenobiotics by cytochrome P450 | 930.2308 | 1189.824 | 0.001539 | 0.014275 |
| General function prediction only | 34828.85 | 34008.47 | 0.001616 | 0.01449 |
| Bacterial secretion system | 7881.923 | 8278.529 | 0.001777 | 0.014934 |
| Tyrosine metabolism | 3763.731 | 4017.412 | 0.001745 | 0.014934 |
| Arginine and proline metabolism | 9600.077 | 10069.35 | 0.001951 | 0.015686 |
| Selenocompound metabolism | 3749.885 | 3610 | 0.001983 | 0.015686 |
| Alzheimer's disease | 1177.308 | 1338.824 | 0.002068 | 0.015894 |
| Sporulation | 329.6538 | 249.2941 | 0.002151 | 0.016072 |
| Prenyltransferases | 4042.423 | 3716.353 | 0.002541 | 0.017527 |
| Biosynthesis of ansamycins | 699.5385 | 632.8235 | 0.002538 | 0.017527 |
| Meiosis - yeast | 79.5 | 50 | 0.002436 | 0.017527 |
| Glycan biosynthesis and metabolism | 918.4231 | 1078.235 | 0.002649 | 0.017815 |
| Aminoacyl-tRNA biosynthesis | 13925.92 | 13551.47 | 0.002759 | 0.018104 |
| Glutathione metabolism | 3662.846 | 3905 | 0.002991 | 0.01916 |
| Secretion system | 15674.88 | 17347.76 | 0.003242 | 0.020284 |
| DNA replication | 7739.808 | 7475.176 | 0.003376 | 0.02064 |
| Nucleotide excision repair | 4278.846 | 4028.941 | 0.003957 | 0.022648 |
| Glutamatergic synapse | 627.6538 | 548.0588 | 0.0038 | 0.022648 |
| Bisphenol degradation | 299.3077 | 249.4706 | 0.003949 | 0.022648 |
| Terpenoid backbone biosynthesis | 6329.962 | 5976.118 | 0.004116 | 0.023066 |
| Lysine biosynthesis | 7203.5 | 7320.412 | 0.004626 | 0.025396 |
| Phosphatidylinositol signaling system | 1466.808 | 1621.471 | 0.004811 | 0.025881 |
| Fatty acid biosynthesis | 5706.115 | 6109.471 | 0.005157 | 0.027202 |
| Ascorbate and aldarate metabolism | 853.5769 | 735.7647 | 0.005399 | 0.027931 |
| Linoleic acid metabolism | 183.3462 | 141.3529 | 0.005595 | 0.028398 |
| Systemic lupus erythematosus | 136.8846 | 175.8235 | 0.006512 | 0.03244 |
| Huntington's disease | 989.6538 | 1141 | 0.007576 | 0.037053 |
| Lipid biosynthesis proteins | 7135.577 | 7439.824 | 0.007752 | 0.037237 |
| Ribosome | 28382.88 | 27323.29 | 0.009435 | 0.043757 |
| Novobiocin biosynthesis | 1312.462 | 1383.824 | 0.009432 | 0.043757 |
| Amino acid metabolism | 1515.538 | 1400.412 | 0.009779 | 0.044583 |
| Membrane and intracellular structural molecules | 8105.577 | 8654.353 | 0.010585 | 0.047458 |
| alpha-Linolenic acid metabolism | 155.2308 | 190.5294 | 0.010872 | 0.047944 |
